# Role of Clinical, Biochemical, and Imaging Parameters in predicting the Severity of Acute Pancreatitis

**DOI:** 10.5005/jp-journals-10018-1202

**Published:** 2017-05-05

**Authors:** Dina Zerem, Omar Zerem, Enver Zerem

**Affiliations:** 1 Medical Faculty, University of Tuzla, 75000 Tuzla, Bosnia and Herzegovina; 2 Department of Medical Sciences, The Academy of Sciences and Arts of Bosnia and Herzegovina, 71000 Sarajevo, Bosnia and Herzegovina; 3 Department of Gastroenterology, University Clinical Center Tuzla, 75000 Tuzla, Bosnia and Herzegovina

**Keywords:** Acute pancreatitis, Clinical outcome, Complications, Computed tomography, Conventional transabdominal ultrasound, C-reactive protein.

## Abstract

**Aim:**

The assessment of the severity of acute pancreatitis (AP) is important for proper management of the disease and for its prognosis. The aim was to correlate clinical, biochemical, and imaging diagnostic parameters and evaluate their prognostic values in the early assessment of severity of AP.

**Materials and methods:**

We prospectively studied 128 consecutive patients with AP. The predictors were clinical, biochemical, and imaging diagnostic parameters. The outcome measure was the occurrence of complications. Abdominal sonogram, contrast-enhanced computer tomography, and pancreatitis-specific clinical and laboratory findings were done.

**Results:**

According to the Atlanta classification, 84 patients (65.6%) had mild and 44 (34.4%) had severe AP. The severity markers were significantly different between the mild and the severe groups (p < 0.001). Leukocyte count, serum albumin level, C-reactive protein (CRP), Ranson, acute physiology and chronic health evaluation II (APACHE II), and Glasgow score were the factors associated with radiological severity grade. Leukocyte count, CRP, Ranson score, APACHE II, and Glasgow score were the factors associated with the number and appearance of acute fluid collections (AFCs). A significant association was found between the number of AFCs and the occurrence of complications [odds ratio 4.4; 95% confidence interval 2.5-7.6]. Hospital stay was significantly longer in the group with severe disease as compared with the group with mild disease (p < 0.001).

**Conclusion:**

Clinical, biochemical, and imaging diagnostic parameters are related to the clinical course of AP and they can predict its severity. This allows us to determine the severity of the disease and to target the patients with high scores for close monitoring and more aggressive intervention.

**How to cite this article:** Zerem D, Zerem O, Zerem E. Role of Clinical, Biochemical, and Imaging Parameters in predicting the Severity of Acute Pancreatitis. Euroasian J Hepato-Gastroenterol 2017;7(1):1-5.

## INTRODUCTION

The course of acute pancreatitis (AP) is highly variable in clinical presentation and its severity. In the majority of patients, the course is mild and can be resolved spontaneously, but in about 20% of patients it may progress to a severe necrotizing form with organ failure and mortality of up to 10 to 50%. Because of this potential for deterioration and fatal outcome, the stratification of the severity of AP is essential.^1-4^

Various methods have been used for predicting the severity of AP and its outcome, such as clinical evaluation, imaging evaluation [contrast-enhanced computed tomography (CECT), magnetic resonance imaging (MRI), and contrast-enhanced ultrasound (CEUS)], and testing of various biochemical markers.^5-15^

Imaging methods have contributed significantly to the staging of severity and prognostic assessment of AP. The most common imaging method of staging of AP is based on CECT. Abdominal CECT scan has been used to determine the degree of severity, extent of necrosis, fluid collections, pseudocysts, abscesses, and prognosis of clinical outcome of AP.^15-19^ The classification by Balthazar et al^9^ is the reference grading system that has been used internationally, because it has shown good correlations with the clinical course and outcome of the disease. In cases where CECT is contraindicated or associated with complications, MRI is an alternative method in the imaging of the pancreas,^15,20^ whereas CEUS has the potential to become a reliable alternative to CECT for assessing the severity of AP and prediction of its outcome.^21,22^

Ranson developed a grading system for AP severity based on clinical and biochemical findings.^6^ The severity scoring system for AP named as the acute physiology and chronic health evaluation (APACHE II) was applied by Larvin and McMahon.^23^ The acute-phase reactant C-reactive protein (CRP) is the best established and most available predictor of inflammation.^3,23^

However, AP is a very complex disease, and despite the existence of several criteria, it is not an easy task to predict its subsequent course. We conducted this study to assess the predictive value of clinical, biochemical, and imaging parameters in the early assessment of severity and outcome of AP.

## MATERIALS AND METHODS

All consecutive patients admitted to our hospital between March 2006 and March 2011, with AP and onset of pain of less than 72 hours before admission, were included in the study. The diagnosis of AP was based on typical symptoms, including acute abdominal pain and a serum amylase level that was three times higher than the reference limit. Informed consent was obtained from each patient on the day of admission. This study was approved by the local ethics committee.

All patients underwent the following examinations: (1) Pancreatitis-specific clinical and laboratory tests; (2) CECT between the 3rd and 5th day following admission, and 30 days after the admission; (3) abdominal sonogram on admission, every day during hospital stay and 10 and 30 days after the admission in patients who were discharged from the hospital earlier.

All patients were assessed by clinical examination and laboratory data. Serum amylase and lipase levels, and CRP were tested and measured at admission, and again at 24, 48, and 72 hours post admission. The pancreatitis-specific clinical Ranson, APACHE II, and Glasgow scores were calculated at the same time. Age, body mass index, ethyology, and length of hospital stay were also monitored.

Abdominal CECT was performed to assess the degree of pancreatic and peripancreatic inflammation, necrosis, and pancreatitis-related fluid collections within the first 5 days. Contrast-enhanced CT was performed using 5 mm axial slices before and after contrast material injection with a spiral CT device (Somatom Sensation 16; Siemens, Erlangen, Germany).

Upper abdominal ultrasound scanning was performed transabdominally using conventional B-mode ultrasound (Logic 400 machine and 3.5 MHz curvilinear transducer, General Electric, Chicago, Illinois, USA), with particular observation of the size and the echogenicity of pancreas and the peripancreatic tissue, the pancreatic and bile ducts. Attention was also paid to peripancreatic acute fluid collections (AFCs), including the lesser sac, anterior pararenal spaces, posterior pararenal spaces, and peritoneal cavity. The size of the pancreas, the number of AFC, as well as any regression or resolution in AFC were recorded.

The criteria for the severity of AP were based on the Atlanta classification, including the presence of local (pancreatic necrosis, pseudocysts, abscess) and systemic complications (e.g., sepsis, organ failure, shock), and according to pancreatitis-specific clinical, imaging, and laboratory findings. According to Ranson and Glasgow score, 0 to 2 characterizes mild AP and 3 or more severe AP. According to APACHE II score, 0 to 8 characterizes mild AP and 9 or more severe AP. The levels of CRP higher than 150 mg/L were considered indicative of severe inflammation. According to Balthazar’s criteria, A, B, and C grade are classified as mild and D and E grade as severe AP. The sensitivity, specificity, accuracy, positive predictive values, and negative predictive values of all parameters were calculated in order to evaluate their diagnostic capacity in identifying the severity of pancreatitis.

Statistical analyses were performed using MedCalc for Windows, version 12.1.3.0 (MedCalc Software, Mariakerke, Belgium). In order to test the differences between the groups in quantitative variables, one-way analysis of variance and Kruskal-Wallis tests were done, depending on the type of data distribution. Also, chi-square test for trend was done in order to test the differences in qualitative variables. Each result was calculated as a mean (median) value and standard error. A p-value less than 0.05 was considered a statistically significant result.

## RESULTS

A total of 128 AP patients were included in this study. Eighty-five patients (66.4%) were males, and the mean age of the patients was 50 ± 12 years. Fifty-nine patients (46%) showed alcoholic pancreatitis and there were 42 (33%) biliary pancreatitis patients. The biliary pancreatitis patients’ mean age was older than that of alcoholic pancreatitis patients (p < 0.05). The other causes of AP were hypertriglyceridemia (11 patients, 9%), idiopathic pancreatitis (11 patients, 9%), and trauma (5 patients, 4%). According to the Atlanta classification, we classified 84 patients (65.6%) as having mild AP and 44 (34.4%) as having severe AP. The characteristics of the patients are shown in Table 1.

All 84 patients with acute mild pancreatitis have shown complete resolution with conservative treatment. The mean length of hospitalization was 18 days (7-84). Hospital stay was significantly longer in the group with severe disease as compared with the group with mild disease (p < 0.001) (Table 1). The severity markers, which were used in this study, were found to be significantly different between the mild and the severe groups (p < 0.001) (Table 1). The mean interval between the onset of symptoms and admission was 35 ± 18 hours.

According to Balthazar’s criteria, we classified 69 patients (54%) as having mild AP and 59 (46%) as having severe AP. Leukocyte count, serum albumin level, CRP, Ranson, APACHE II, and Glasgow score were the factors associated with the radiological severity grade in our study (Table 2).

Using the transabdominal conventional B-mode ultrasound study, we identified 61 patients (47.6%) as having AFC and 67 patients (52.4%) without AFC. Leukocyte count, CRP, Ranson, APACHE II, and Glasgow score were the factors associated with the number and appearance of AFCs in our study (Table 3).

A significant association was found between the number of AFC and the complication occurrence [odds ratio (OR) 4.4; 95% confidence interval (CI) 2.5-7.6]. Multivariate model adjusted for age and Ranson score is presented in Table 4. Cut-off point of >1 AFC was found to be the prognostic factor for complications.

**Table 1:** **Table 1:** Relation between severity of AP and clinical, imaging, and biochemical parameters

		*Grade of severity of AP*					
		*Mild (n = 84)*		*Severe (n = 84)*		*p-value*	
Gender†							
Males		56 (66.7%)		29 (65.9%)		>0.05	
Females		28 (33.3%)		15 (34.1%)			
Age (years)		49 ± 12		50 ± 15		>0.05	
Hospital stay (days), mean		10.2		22.6		<0.001	
S-amylase (IU/L)		938 ± 335		1088 ± 248		>0.05	
APACHE II score†							
≥9		9 (10.7%)		40 (90.9%)		<0.0001	
<9		75 (89.3%)		4 (9.1%)			
Ranson score†							
≥3		8 (9.5%)		40 (90.9%)		<0.0001	
<3		76 (91.5%)		4 (9.1%)			
Glasgow score†							
≥3		9 (10.7%)		39 (88.6%)		<0.0001	
<3		75 (89.3%)		5 (11.4%)			
CRP†							
≥150		11 (13.1%)		3 (6.8%)		<0.0001	
<150		73 (86.9%)		41 (93.2%)			
CTSI†							
≥7		15 (21.4%)		31 (70.5%)		<0.02	
<7		69 (78.6%)		13 (29.5%)			
Number of AFC†							
≥2		14 (16.7%)		32 (72.7%)		<0.01	
<2		70 (83.3%)		69 (27.3%)			

†Number of patients with mild/severe scores

**Table 2:** **Table 2:** Univariate analysis of CT Balthazar grade and biochemical parameters

		*Balthazar grade*											
*Parameter*		*A*		*B*		*C*		*D*		*E*		*p-value*	
Leukocytosis		9		8		12		11		19		0.0001	
AST (IU/L)		78		86		87		122		76		0.21	
LDH (IU/L)		76		77		112		154		130		0.08	
T bilirubin (mg/dL)		16		15		18		14		17		0.07	
Albumin (g/dL)		36		35		34		32		26		0.001	
S-amylase (IU/L)		1,650		1,662		1,650		1,270		1,870		0.13	
Ranson score		2		2		3		4		5		0.0001	
Glasgow score		1		1		3		3		4		0.0001	
APACHE-II score†		4		5		9		12		19		0.0001	
APACHE-II score‡		2		3.5		8		11		17		0.0001	
CRP (mg/dL)†		22		34		87		132		345		0.0001	
CRP (mg/dL)‡		11		22		61		111		325		0.0001	

†Value of parameter at admission; ‡value of parameter after 72 hours; AST: Aspartate aminotransferase; LDH: Lactate dehydrogenase

## DISCUSSION

Various methods have been used to predict the progress of AP, such as clinical evaluation, imaging evaluation, and testing of various serological markers.^10-15^ In our series, we investigated the correlation between the changes of the clinical predictors, pancreatic enzyme, the biochemical markers, and the results according to the Balthazar CT grade and number and appearance of AFCs obtained by ultrasound examinations. In our previous study,^24^ regarding these series, we showed that the presence and number of AFCs diagnosed by CTUS at the beginning of AP was correlated with the clinical course of the disease, complications, and mortality. In this study, we present some other aspects of the correlation among clinical, biochemical, and imaging parameters and evaluate their prognostic value in the early assessment of severity and outcome of AP.

**Table 3:** **Table 3:** Univariate analysis of number and appearance of AFCs as predictive factors and biochemical parameters

		*Number and appearance of AFC*											
*Parameter*		*0*		*1*		*2*		1*		2*		*p-value*	
Leukocytosis		9		10		11		13.5		19		0.0001	
AST (IU/L)		85		91		95.5		92.5		70.5		0.74	
LDH (IU/L)		76		87		126		91		94		0.07	
T bilirubin (mg/dL)		15		18		18		17		17		0.08	
Albumin (g/dL)		35		34		34		35		32		0.07	
S-amylase (IU/L)		1650		1650		1330		-		1870		0.3	
Ranson score		2		2		2		-		5		0.0001	
Glasgow score		1		2		2		1		4		0.002	
APACH-II score†		4		8		8		5		20		0.0001	
APACH-II score‡		3		8		7		3		17		0.0001	
CRP (mg/dL)†		24		78		86		94		327		0.0001	
CRP (mg/dL)‡		14		46		56.5		56.5		278		0.0001	

*Heterogeneous appearance of AFC; †value of parameter at admission; ‡value of parameter after 72 hours; AST: Aspartate aminotransferase; LDH: Lactate dehydrogenase

**Table 4:** **Table 4:** Prediction of complications based on AFCs

*Predictors*		*Units of increase*		*Adjusted OR*		*95% CI*	
Acute fluid		1 AFC		3.2		1.72-6.05	
collections				1.0 (ref.)			
Age		1 year		1.01		0.96-1.05	
				1.0 (ref.)			
Ranson score		Severe mild		1.61		1.05-2.48	
				1.0 (ref.)			

The majority of patients with AP have mild disease (66% in our series), and their clinical symptoms and laboratory findings resolve with supportive care within a few days. However, in about 20% of patients, the disease can progress to a severe necrotizing form with organ failure and local complications, such as necrosis, abscess formation, and pseudocysts with mortality of up to 10 to 50%.^1,4,25^ According to the Atlanta classification and Ranson criteria, we classified 44 patients (34.4%) as having severe AP. We explained such high percentage of severe form of AP by the fact that 30 patients were transferred from other hospitals (between days 2 and 17 after the onset of pain) and enrolled in the study. All 30 patients transferred from other hospitals presented with severe form of AP.

In this study, most patients were of an alcoholic and biliary origin. Similar to other studies,^1,2,26^ we reported that the serum amylase and lipase concentrations were higher in the patients with biliary pancreatitis than the patients with alcoholic origin of AP. Also, the biliary pancreatitis patients were older than the patients with alcoholic origin of AP, but any other differences among clinical, biochemical, and imaging parameters were not present.

In our series, the results of the imaging methods (CT and ultrasound) were prospectively evaluated and compared with the scores based on physiologic and health evaluation system (Ranson score, Glasgow score, APACHE II score), and biochemical parameters (CRP, leukocyte count, aminotransferases, serum albumin level, serum amylase level) with intention to predict the severity of AP.

Various scoring systems are used for predicting the severity of AP.^1,27,28^ The APACHE II, which is a nonspecific scoring system, has been in use for patients with AP since 1989.^23^ The score is the sum of various physiological parameters. This system is complex, difficult to perform, and has a low accuracy rate for identification of local complications.^29^ However, previous studies have shown that AP patients with high APACHE II score were likely to have a severe course of the disease.^1,4,27^ In our series, the mean APACHE II score was significantly higher in the severe pancreatitis group (Tables 2 and 3). This confirms its high specificity and sensitivity in predicting the clinical course of AP.

In our study, Glasgow and Ranson scores have shown significant correlation with the imaging assessment [computer tomography severity index (CTSI) and AFCs diagnosed by ultrasound] and with the clinical course of the disease (Tables 2 and 3).

Serum CRP is an acute-stage protein, i.e., synthesized in the liver. It is elevated in various inflammatory conditions, and serves as a nonspecific inflammation marker. This parameter is usually used because it is simple and cheap.^3,23^ Also, CRP is a proven predictor of severity for AP when serum level of over 150 mg/L is measured within 48 hours after the onset of symptoms.^13,27,30^ Our results show statistically significant higher serum concentrations of CRP in patients with severe disease. Also, changes of the CRP level during the treatment reflect the disease prognosis. In our study, the CRP titer was a predicative factor with good correlation to the radiological grade on multivariate and univariate analysis.

In our study, leukocyte count was the factor that was associated with the radiological severity grade and the number and appearance of AFC. Serum albumin level was the factor associated with the radiological severity grade, but not with the number and appearance of AFC (Tables 2 and 3).

After the adjustment for age and Ranson score in multivariate model, AFC remained prognostic for complications. An increment in 1 AFC resulted in 3.2 higher chances of complications, and cut-off point of >1 AFC was prognostic for their occurrence with high sensitivity and specificity (Table 4).

In conclusion, our study suggests that the results of the findings based on imaging methods correlate well with the scores based on physiological and health evaluation system, and with the biochemical inflammatory parameters during the clinical course of AP. This allows us to determine the severity of the disease and target the patients with high scores for close monitoring and more aggressive intervention.
